# Smart City and Crisis Management: Lessons for the COVID-19 Pandemic

**DOI:** 10.3390/ijerph18157736

**Published:** 2021-07-21

**Authors:** Mahnoosh Hassankhani, Mehdi Alidadi, Ayyoob Sharifi, Abolghasem Azhdari

**Affiliations:** 1 School of Planning and Design, Iran University of Science and Technology, Tehran 1684613114, Iran; mahnoush.hasankhani@gmail.com; 2 Faculty of Arts and Architecture, Tarbiat Modares University, Tehran 1411713116, Iran; m.alidadi@modares.ac.ir (M.A.); abolghasem.azhdari@modares.ac.ir (A.A.); 3 Graduate School of Humanities and Social Sciences & Network for Education and Research on Peace and Sustainability (NERPS), Hiroshima University, Hiroshima 739-8511, Japan; 4 Graduate School of Advanced Science and Engineering, Hiroshima University, Hiroshima 739-8511, Japan

**Keywords:** COVID-19, urban resilience, community well-being, crisis management, smart city

## Abstract

COVID-19 shocked cities around the world and revealed the vulnerability of urban lives and functions. Most cities experienced a catastrophic disturbance that has lasted for a long time. Planning plays a critical role in responding efficiently to this crisis and enabling rapid functional recovery in the post-disaster era. Cities that have implemented digitalization initiatives and programs are likely to have more capacity to react appropriately. Specifically, digitalized cities could ensure the well-being of their residents and maintain continuity of urban functions. This research aims to analyze the role of technology in crisis management in the last two decades and provide appropriate policy recommendations for dealing with the COVID-19 pandemic. Systematic literature review and subjective content analysis are employed to investigate the effects of technology on community well-being and making cities more resilient in past crises. This study shows that different technology-driven policies and actions enable crisis management, enhance community well-being, and increase urban resilience. Technology has enhanced coping and recovery capacities by increasing participation and social connectedness, enhancing physical and mental health and maintaining the functionality of education and economic systems. These have been achieved through various solutions and technologies such as social media, telehealth, tracking and monitoring systems, sensors and locational applications, teleworking systems, etc. These solutions and technologies have also been used during the COVID-19 pandemic to enhance community well-being and sustain urban functions. However, technology deployment might have adverse effects such as social exclusion, digital divide, privacy and confidentiality violation, political bias and misinformation dissemination, and inefficient remote working and education. It is suggested that to mitigate these side effects, policymakers should liberate the process of digitalization, increase the accessibility to digital services, and enhance digital literacy.

## 1. Introduction

Uncertainty, unpredictability, and complexity are prominent features of urban life in the 21st century [1]. Due to the fast pace of global changes and some unforeseen universal crises (such as the COVID-19 pandemic), uncertainties have increased [2], and cities have become more vulnerable [1], specifically in terms of the quality of life and well-being of residents [3,4]. Indeed, risks or disasters have become an undeniable part of the urbanization process [5]. Irrespective of differences in terms of magnitude, and groups and places that they affect, disasters cause infrastructure damage and human-related sufferings [6]. Therefore, there is a need for collective actions and decisions that reduce hazard risk and vulnerability of people, infrastructures, and places [7]. Accordingly, lowering urban vulnerability is one of the main areas on which planning actions and processes focus. Through various trajectories such as rational application of knowledge, improved learning process, and bargaining processes to mobilize actors and stakeholders, planning can play different roles in responding to crises [8]. However, there is no straightforward planning approach and strategy to find proper ways of dealing with disturbances and unpredictable situations. Learning by doing [9] is a pragmatic strategy that equips cities and authorities with incremental experiences to deal with problems. In the past few years, urban resilience thinking has guided urban planning efforts to reduce vulnerabilities and increase the efficiency of processes to cope with disturbances [10]. In other words, urban resilience is a response to changes and disturbances by increasing the planning, absorption, and recovery capacities of the system on the one hand and adaptation capacity to transform to new normal states on the other hand [1,11,12,13].

One of the main planning tools to reduce the risks and vulnerability of populations is using technology to increase preparedness and recovery capacities in the face of crises. In the urban planning domain, such planning tools, efforts, and initiatives are often referred to as smart city solutions [14]. A smart city has three main pillars, including technology, people and institution. Using technology in crises management requires technology deployment along with empowering and engaging people through institutions [15]. The efficiency of the smart city approach is highly advocated by researchers, planners, and practitioners [16,17]. Indeed, empirical studies have shown that smart technologies have been massively used in crises management. Specifically, smart city solutions are increasingly considered essential for enhancing the well-being of residents and communities in urbanized countries [18]. Since the emergence of COVID-19, the necessity of deploying technology to improve community wellbeing and maintain urban functions has increased [19,20]. As this research focuses on the effect of technology deployment on community well-being, it should be clarified what we mean by well-being.

The term well-being refers to various abstract and objective meanings that philosophically determine an individual’s living space and conditions. These meanings could be different according to the aims and objectives of the researcher or actor [21,22]. Regardless of the multiplicity of well-being’s meanings in the literature [23,24,25,26], in this study, we focus on specific parts of well-being that could be improved by technology [24,27,28,29]. Another issue is that due to the diversity of policymakers and stakeholders, urban planning, has limited capacity for intervention. So, through a review of the literature, we found three main areas of community wellbeing that the smart city approach could improve. 1. Participation, transparency, and social connectedness of residents, 2. Healthcare of residents, both physically and mentally, 3. Education of people and the employment of residents. Additionally, we discussed how technology has contributed to improving these areas during the COVID-19 pandemic. Overall, this article discusses how smart city solutions and technologies can enable cities to better prepare for and respond to future similar disasters (such as the COVID-19 pandemic). We will assess and analyze lessons from technology-driven urban planning systems and discuss their utility for enhanced response to the pandemic. We further elaborate on the roles that smart city technologies can play to deal with disasters and promote the well-being of citizens in the face of the COVID-19 pandemic.

## 2. Research Design

The main objective of this study is to analyze and understand how technology has been used as an instrument to improve the well-being of citizens during recent crises. The main questions guiding this literature review are how has technology been used to increase participation, transparency, and social connectedness in previous crises? How has technology been used to protect the physical and mental health of residents in previous crises? How has technology been used to keep urban functions and sustain education in previous crises? and how has technology deployment been addressed as an instrument to cope with associated problems of COVID-19? We will use inductive and deductive qualitative content analyses using scientific articles and textbooks published in five selected databases [30]. After determining different aspects of well-being and technology, we will explore the use of technology in the COVID-19 crisis in the main areas mentioned above. In the final stage, we will outline the challenges and barriers of using technology in crisis management, provide pathways for future research, and discuss policy implications.

### Literature Review Method and Procedure

**Figure 1:**
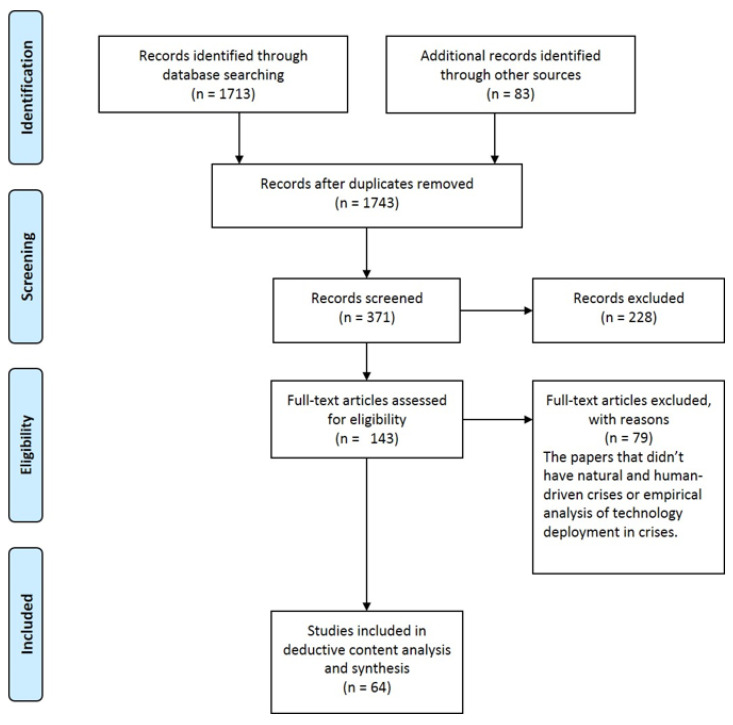
The PRISMA diagram showing various steps taken for the purpose of this systematic review [34].

The systematic literature review is one of the common methods of investigating and analyzing the literature that includes six main steps as shown in Figure 1: literature search protocol design, literature search, screening, synthesis, analysis, and reporting [31]. The three first stages deal with finding and gathering data, and the last three are about using these data for theoretical and empirical contributions. Content analysis is an approach in the systematic literature review [32] that is suitable for interdisciplinary areas, where a concept or subject is studied in various disciplines [33].

In the first step, we used inductive content analysis to find the main areas of well-being and urban functionality that could be affected by the technology or smartness of a city. Then we determined four main questions that should be answered through a systematic literature review. The primary data needed to answer the research questions can be collected from different sources including published papers, reports, books, and newspaper articles. For systematic literature review and deductive qualitative content analysis, we conducted broad searches using six internationally recognized scientific: databases: Science Direct, PubMed, Springer, Sage, Taylor and Francis, and MDPI. In the fields of urban planning and urban studies, these databases are widely known for publishing quality peer-reviewed research. The search string in this study was chosen broadly, including “smart city OR technology OR digitalization” AND “crisis management OR disaster OR pandemic.” The search is only based on documents and cases published in English. We found 1713 documents in databases and 83 other documents from Researchgate, that 53 of them were duplicates. We first manually screened the titles of the retrieved documents and 371 papers that were relevant were selected for detailed screening by reading the abstracts. The main criteria for screening were to have issues related to technology deployment or smart city under crisis situations discussed in the titles. The same criteria were also applied for screening the abstracts. After screening the abstracts, 228 papers were excluded as they were irrelevant. Papers that did not include empirical evidence on the deployment of technology for better crises management and for enhancing community well-being were excluded. We assessed 144 full texts and scrutinized the content of these documents. As we looked for papers with empirical analysis and case studies of crises in previous years, we just chose the articles with the empirical analysis of technology in a specific disaster before the COVID-19 pandemic. As there was a limited number of studies that empirically have analyzed technology deployment during COVID-19 (when this paper was designed and drafted), papers that just mentioned case studies were also included. We finally reviewed 64 papers.

## 3. Analysis and Synthesis of the Literature

### 3.1. Smart City as a Planning Tool during Crises

Digitalization or smart city approaches strengthen urban resilience through risk reduction strategies and practices [35]. The smart city is known as an adaptive urban planning approach to cope with disturbances [36,37,38]. While there is no consensus regarding the definition, principles, objectives, and different aspects of a smart city [39,40,41,42,43], the smartness of cities includes three main elements: people, institutions, and technology [15,44]. During crises, smart city initiatives enabled by Information and Communication Technologies (ICTs) play critical roles in responding and recovering efficiently and improving learning by doing [45,46]. For example, as a tool, social media plays a critical role in mobilizing actors, communities, and resources [38]. Authorities also can use social media through big data analytics to make more democratic decisions and develop strategic solutions for disaster responses [47]. There are several real-world cases in which governments have used social media in crisis management to have more practical, updated, and democratic responses [48]—for example, using Wikis as a collaborative tool [49]. As cities massively use technology during crises to cope with associated problems [27,50], this research focuses on this aspect of the smart city. Using technology in cities has two main functions: data and service provision [42,43]. Technology provides different data types, satellite images, location-based applications on mobile phones, tracking devices, sensor-based information in the city, social media information, etc., to increase urban resilience. Additionally, technology helps to provide services in shopping, healthcare, businesses, education, social relations, and so on [51]. It also increases the profitability of investments, enhances resiliency and adaptation capacity, and improves dwellers’ quality of life [42,43]. Improving the well-being of residents and community planning systems can, in turn, increase the resilience and crisis response/recovery capacity [52,53,54].

Sustainable Development Goals (SDGs) have also emphasized different issues related to urban life [55], such as leveraging technology to improve citizens’ well-being and the urban digital infrastructure [36,37]. While there are some arguments regarding the negative impacts of technology-centered urban planning strategies compared to human-centered ones, digitalization is increasingly gaining prominence in urban areas and crisis management [18]. The COVID-19 has, in particular, showed the necessity and benefits of such digitalization processes and how [55]. Developing ICTs in cities can particularly increase people’s well-being in urban areas if it facilitates inclusive accessibility to services [51].

### 3.2. Participation, Transparency, and Social Connectedness during Crises

Community engagement is a central issue in the Sendai Framework for Disaster Risk Reduction to prepare residents and mitigate risks during and after disasters [56]. Technology can increase social capital and civic participation for making disaster management more efficient [57]. On the other hand, technology can enhance transparency that contributes to enhanced trust between residents and governmental bodies. During the last few years, by developing ICT infrastructures, local governments have provided opportunities to enhance public participation through social media. Moreover, along with transparency and accountability of governmental processes, smart city initiatives empower citizens through active engagement [58,59].

Social media has been used as a new communication method during crises [60]. Communication enabled by social media, for instance, played an unprecedented role in the Great East Japan Earthquake. Cheng, Mitomo, Otsuka and Jeon [57] analyzed the effectiveness and different aspects of crisis management tools enabled by social media. They showed that despite two types of active and passive use of social media, there was a positive effect of ICT on the recovery process through making networks, enhancing bonding social capital, and increasing participation. Kankanamge, et al. [61] showed that posts with images on Facebook and Twitter by emergency organizations in Australian states increase people’s attention to prepare more and enhance their involvement in making decisions and taking actions. Accordingly, during the tropical Cyclone Winston in Fiji (2016), Facebook and Twitter, as the most common forms of social media, played critical roles in linking people to the recovery process [60].

Bird, et al. [62] analyzed the importance of social media during flooding in Victoria, Australia, and concluded that Facebook and Twitter were productive tools for disseminating informal information. They discussed that there might be inaccuracy regarding information reported by ordinary people, but social media administrators recognized and corrected inaccurate information. Additionally, they showed that social media facilitated better communication and empowered citizens to take emergency actions. However, as the accuracy of information is an essential issue, people follow warnings and information from authentic media and sources rather than social media. The result of a study by Boas, et al. [63] in the case of Typhon Meranti in Xiamen, China, showed that people during catastrophic disasters rely on authentic information from the government, albeit they may get this information from formal channels of government in the social media. Linders [64] discussed three types of relationships in social media; citizen-to-citizen (C2C), government-to-citizen (G2C), and citizen-to-government (C2G), while Rajput, et al. [65] also discussed the inter-governmental social media-based collaborations. Based on Linders [64], the connection between people is an important issue that can enhance the well-being of residents. Boas, Chen, Wiegel and He [63] concluded that in China, during disasters, the usefulness of social media is for C2C communication. Kitazawa and Hale [66] analyzed the effectiveness of social media for disaster management during Kanto–Tohoku Typhon 2015 in Japan. They argued that social media makes people more attentive to warnings and enhances their awareness. Further, social media information may make people more interested in preparation and response actions. Similar results are reported by Fang, et al. [67] for the case of the 2016 Typhon in Wuhan, China.

### 3.3. Physical and Mental Health of Residents and Community during Crises

Disasters threaten people’s well-being and health along with damaging infrastructures [7,68,69]. Pandemics could be considered as disasters that massively affect community health and healthcare systems [70]. Undoubtedly, addressing issues related to the health of citizens during disasters should be prioritized as it may have long-lasting effects on community well-being. Additionally, the Sendai Framework for Disaster Risk Reduction (2015) has emphasized the health and well-being of citizens and highlighted their significance [5]. Technology deployment for enhancing the efficacy and efficiency of the healthcare system, known as E-health, can improve disaster management. In this respect, different aspects of E-health could be improved through the digitalization of processes by using electronic health records, mobile health, augmented reality, artificial intelligence, big data, and other solutions. They increase the effectiveness and inclusiveness of healthcare services delivery during and after disasters. Disaster E-health is a new area of study that emerged in public policy and combines disaster management, disaster medicine and E-health [71,72]. Telemedicine, for example, is a service that can ensure that people stuck amid disasters can receive remote services through telecommunication networks [73]. Due to the increasing vulnerability of cities and rural areas to natural and human-driven disasters, telemedicine or E-health has gained more currency in the last decade.

A pilot telemedicine project was implemented in Prayag city in India to reduce the spread of cholera. The project used teleconsultation, telemonitoring of public health and health care facilities, and telehealth education. Additionally, this pilot project processed the gathered data to prevent the spread of the disease. Ayyagari, et al. [74] analyzed the effectiveness of telemedicine in this project and concluded that it has successfully prevented the spread of the disease in less than a month. In the 2015 California Valley Fire, telemedicine played a critical role in the first days of the disaster and the following weeks. People used different tech devices to contact doctors remotely and report their physical and mental problems.

Similarly, Pasipanodya and Shem [73] investigated the effectiveness of this program on spinal cord injury and discussed how it had helped healthcare delivery through video and audio reports. Telehealth also was applied in Hurricane Sandy that had a widespread effect on the well-being and health of residents in NYC in 2012. It included various functions such as clinical video telehealth, home telehealth, and transmission of diagnostic images and other patient data for post-disaster healthcare delivery. Evaluation of this program by Der-Martirosian, et al. [75] showed that the number of people who have used telehealth for triage increased substantially while mental health issues moderately increased compare to pre-disaster situations.

Mental health problems are also a long-lasting effect of disasters. In the last decade, healthcare systems have used technology to lower these effects. For instance, the 2010 Haiti earthquake caused about 300,000 deaths and more than 1.5 million injuries and displacement. Augusterfer, et al. [76] analyzed how telemental health services contributed to improving the well-being of residents after this disaster. The most important outcome of implementing this policy was connecting Haiti to the rest of the World. Medical schools around the World provided triage and consultation through satellite telephones and video calls. Telepsychiatry has more potential than telemedicine to be provided remotely [77]. In this respect, telepsychiatry was implemented in Pakistan during different earthquakes that happened after 2010. Qadir, et al. [78] investigated different aspects of these services and found that low-income people exposed to disasters take their mental health problems for granted. Additionally, as there are limited places and services for mental health remedies and consultations in their vicinity, telepsychiatry played a critical role in post-trauma recovery.

### 3.4. Education and Employment during Crises

E-learning, remote working, and remote services are common areas that have gained more currency in the last two decades in ordinary days and during disasters. Depending on their magnitude, disasters may have temporary or long-term effects on education systems and employment. However, COVID-19 is a new type of disaster with the characteristics of catastrophic and chronic disasters and has deeply affected local and national economies and education systems [79]. Through one year of COVID-19, more than 1.6 billion students have been affected [80]. As the focus of this section is on previous disasters, we found few cases that governments have used technology for education and employment recovery. On the other hand, this area’s literature mainly focuses on educating people about the subjects related to disasters, not formal education. So, we can review the capacities and capabilities of smart cities to transform their face-to-face education and working conditions to e-learning and remote working.

Emergency remote teaching is a solution for educating people in crisis circumstances by the deployment of technology. These situations need innovative measures to deliver education services to all students regardless of their social and geographical conditions. In South Africa, a private higher education institution that has provided online higher education services has been asked to share its infrastructures to provide services for people exposed to disasters in recent years. It had a blended teaching approach on routine days and extended its programs for emergencies. The institution has two functions: teaching lecturers and then reassuring that students receive education services properly [81]. There are some other forms of chronic issues such as inequality in distributing educational facilities and infrastructures that information and communication technologies could help mitigate these disparities. The Australian government, for example, provided some services for students who live in rural areas and do not have accessibility to metropolitan areas by videoconferencing and blended e-learning. They managed virtual visits to museums, zoos, and natural environments [82].

Disasters also affect working systems during and after their occurrence. While there is a lot of research regarding other aspects of disaster risks, little attention has been paid to the nature, conditions, productivity, and other factors related to jobs. Different concepts such as telework, remote work, and mobile working emerged as areas that ICTs enabled to keep urban functioning [83]. Preparing initiatives and strategies for both disaster and recovery periods is necessary to help the jobs survive. In many ways, businesses would be affected by disasters, such as the damage of infrastructures in floods and earthquakes and low demand for services during the COVID-19 pandemic. In both cases, there is a need for resilience-building strategies. For example, several earthquakes between 2010 and 2011 in New Zealand resulted in many cases of business disruptions and job losses. Most offices lost their places, assets, and infrastructures. Green, et al. [84] investigated two private and public organizations in Christchurch that were highly suffered from the earthquake to know how telework increased their adaptability to disruption and facilitated business continuity. They concluded that public and private organizations increased their resilience through teleworking by improving organizational, personnel, and technical capacities.

## 4. Deployment of Technology in Cities during COVID-19

While humankind has faced different pandemics in the last century, COVID-19 is one of the most influential human-driven disasters that caused deaths and unprecedented disruptions in urban life [20,27]. This challenge has come to a situation that some have called World War COVID to explain the destruction level of the pandemic [85]. Specifically, as COVID-19 has affected all aspects of human life, governments have been forced to impose restrictions to mitigate the negative externalities of the pandemic. There is a critical argument in urban planning on how to manage cities in the wake of the pandemic to maintain the functionality of fundamental urban systems and secure the well-being of residents [86]. Cities are known as the epicenter of COVID-19 [87], mainly because high population density and mobility can increase the infection rate rapidly [88]. Global health governance has struggled to cope with the urbanized characteristics of the pandemic [89]. The necessity of using technology and innovative solutions to deal with the pandemic has been emphasized by all health-related organizations [90]. Earlier, we showed how technology has been used in crisis management in the last two decades to mitigate the effects of disasters on the well-being of citizens. As discussed, while well-being is a broad concept that includes various aspects of human life, in this research the focus is on the areas that are directly affected by technology in urban systems. Technology deployment has been used in three main areas to increase participation: transparency, and social connectedness of residents; to enhance their physical and mental health; and to maintain the functionality of education and employment sectors. In the following sections, an overview of technology deployment to cope with COVID-19 impacts is presented.

### 4.1. Participation, Transparency and Social Connectedness during COVID-19

COVID-19 influenced all personal, social, and political aspects of urban life. So, local authorities and political actors emphasized the deployment of technology to increase the participation of residents in decision-making, particularly regarding COVID-19 decisions, and increase the transparency of these processes. Increasing social connectedness is another objective that is sought through the deployment of technology in cities during COVID-19 (see Table 1). Social media is the most critical and influential technology that has benefitted people and governments to cope with COVID-19. Initially, governments have used social media in the COVID-19 era to interact with people for making decisions and building trust through democratic processes (e.g., the case of Singapore) [91]. Secondly, social media is known as a way of information dissemination to increase preparedness (e.g., Penang city in Malaysia). As can be seen from Table 1 in Asian and African cities, Facebook has been used to communicate between people and government to increase the transparency and trust between governmental bodies and ordinary people. Thirdly, the connection between people living under COVID-19 restrictions is another function of social media in this situation. Fourthly, the information extracted from social media through big data analytics empowers decision-makers to predict the disturbances. In the context of the COVID-19 pandemic, El Azzaoui, et al. [92] analyzed Twitter data and found that by analysis of social media data outbreaks could be predicted about 7 days before authorities formally be informed.

### 4.2. Physical and Mental Health of Residents during COVID-19

Based on the level of vulnerability of communities and the risk level of disasters, residents’ mental and physical health could be affected [93,94,95]. There are millions of people who are mentally and physically suffering from COVID-19 and its consequent restrictions. Smart cities such as New York City (NYC) have more capabilities to cope with the pandemic and its consequences [27]. E-health, telehealth, and telemedicine are examples of smart city solutions to increase the delivery efficiency of healthcare services. In NYC and other cities, as shown in Table 2, to cope with COVID-19 physical and mental problems, different initiatives such as making dashboards that automatically gather data about patients from different sources were implemented. Data sharing systems were also provided in all hospitals to ensure that doctors have accessibility to healthcare processes for all patients. Additionally, emotional and psychological support for patients and healthcare workers became the priority of hospitals by using technology-based solutions to mitigate the adverse well-being effects of COVID-19 [88]. As there were severe limitations regarding visiting patients in hospitals, NYC hospitals provided tablets with video applications to allow patients to get in touch with their families [90]. Elsewhere, various technological functions have been applied to provide efficient healthcare services, including locational tracking systems of citizens (Tel Aviv) [96] to track down contacts of infected patients and to monitor the physical conditions of quarantined patients (Seoul) [97]. Additionally, other functions linked to smart city processes (Table 2) could be mentioned, including (1) simulating safe and high-risk places based on locational tracking and big data (Liverpool and Singapore), (2) using AI to analyze different types of data [98], (3) detecting potentially infected individuals based on thermal camera scanning (Wuhan) [99], (4) promoting dashboards for information sharing (NYC and Dubai), and (5) tracking the mobility of infected people and physical distances to control the spread of the virus (New Castle and Istanbul), etc. [100]. However, the most important technology deployment has been seen in tracking and monitoring residents in public places such as TraceTogether and SafeEntery application in the case of Singapore that the former was voluntary and the latter compulsory [101]. Beyond the practical usefulness of employing technology, new smart devices have also been used to provide pieces of evidence for research. Wearable devices, for example, have been used to analyze the effect of COVID-19 on the physical activity and sociability of residents [102,103].

### 4.3. Education and Employment during COVID-19

One of the main features of this pandemic compared to previous disasters is its influence on education systems and working conditions. From the first months of the pandemic, cities around the world imposed restrictions on gatherings, including schools and universities, and shut down unnecessary jobs. Universities and schools canceled all of their face-to-face teachings from laboratories to regular classes to mitigate the associated risks of the COVID-19 outbreak [104]. Countries and cities have different emergency remote learning strategies from blended to flexible learning. Various technology capacities in cities were employed to educate students, using social media (Facebook, WhatsApp, Telegram, etc.) and mobile applications, for instance [79]. Additionally, different strategies were deployed to connect teachers and students by using video conferencing, interactive whiteboards, and virtual visits of remote sites during courses [82].

Beyond the health impacts of COVID-19, businesses have suffered dramatically, and millions of people have lost their jobs. COVID-19 has made workers and employers change working conditions to protect society and the employees. This situation led to more remote working as a solution to reduce the vulnerability of businesses and maintain their functionality [105]. While teleworking as an initiative was proposed in different disasters in the last decades [83], COVID-19, for the first time, emphasized the necessity of this strategy to keep urban functions. However, the effect of COVID-19 on jobs depends on the nature and conditions of jobs, and those who need face-to-face communications are more vulnerable [106,107]. Cities worldwide have used different technology-driven initiatives to improve the well-being of residents through keeping urban functions. For example, London businesses employed various delivery systems to provide the basic needs of residents’ online shopping. Additionally, they have used sensor-based technologies to evaluate the performance of jobs for stimulation plans (see Table 3). Additionally, in Seoul, the municipality uses big data capabilities to map the high and low-risk areas to have a smart shutdown schedule of businesses.

### 4.4. Effectiveness of Smart City Projects in Managing COVID-19

As discussed in the previous section, technology deployment in different ways has empowered local authorities to control the spread and mortality rates of COVID-19. While COVID-19 is an ongoing crisis and judging the effectiveness of this planning approach is problematic, some recent studies have assessed the benefits of implementing smart city projects for controlling COVID-19. Yang and Chong [108] have quantified the positive effect of smart city projects in different urban areas in China. While they did not present detailed information on the implemented smart city projects, they concluded that the positive effect is more tangible in large cities than in small and medium cities. El Azzaoui, Singh and Park [92] also emphasized the effectiveness of big data analytics applied in the US. They were able to predict the outbreaks of COVID-19 about a week prior to formal confirmation of cases.

One of the significant problems of implementing smart city projects, specifically in less democratic societies, where central government control many aspects of urban life and decision making, is privacy issues. However, since the emergence of COVID-19, the tendency to participate in the research and practice of smart cities has accelerated. The result of research-based projects during COVID has paved the way for the adoption of more technology-based policy-making mechanisms [109].

## 5. Challenges and Barriers of Using Technology in Crisis Management

### 5.1. Privacy, Trust, and Human Rights

Although technology deployment to fight COVID-19 is a ubiquitous solution in cities worldwide, there are serious problems and concerns regarding the trustworthiness of these systems. Governments and corporates use geolocational applications and sensors that collect real-time data. Addressing human rights at the digital level is a critical issue that should be considered in digitalization [110]. Moreover, as people use technology in their everyday lives, the corporates responsible for the digitalization of the city have great accessibility to the internet of things and users’ information that increase privacy concerns. So, the privacy of users, digital right of citizens, ethical promises and confidentiality of patients, false medicine delivery, and unauthorized medical research and experiments are critical concerns that should be appropriately considered for better technology deployment during crises [111].

### 5.2. Inclusiveness

There are concerns that digitalization may increase inequality and social segregation as all people do not have equal access to technology [110]. The digital divide concept in planning literature refers to this; specifically, it emphasizes marginal and socially vulnerable groups [112]. The digital divide can be explained in two levels, first, access to technology, second, the ability to use it appropriately [113]. Specifically, during COVID-19 older people are not familiar with technological devices and are excluded from services provided by the internet [114]. Alongside that, multicultural cities where people may have different local languages may fall behind the participation and lack accessibility to the provided services. At the same time, also, there is a threat of symbolic participation rather than active engagement of society in technology-driven ways of participation [58]. Overall, the experience of COVID-19 showed that inclusiveness of technology use and service delivery is an important issue that needs more attention from scholars and practitioners.

### 5.3. Political Bias and Misinformation Dissemination

The smartness of cities undoubtedly is a positive feature that increases the efficiency of service provision, participation processes, transportation systems, healthcare, etc. However, the digitalization of the city itself may come to action in a nondemocratic process just because it has various benefits [115], while the deployment of technology should be based on the demand and need of local actors. Additionally, politicians and authorities may take advantage of technology as it is an undeniable necessity of urban systems. For instance, non-democratic regimes may see the increased reliance on smart solutions and the increased availability of citizen data as an opportunity to strengthen power relations and further control the free flow of information [20]. Moreover, People may intentionally or unintentionally disseminate wrong information through social media. While social media administrators can recognize and correct them [62], there are also concerns about the political orientations in the strategic perspective of international corporations. They may use their reputation to mislead the public domain and interrupt active and real democracy.

### 5.4. Technical Issues

There are strong concerns regarding the use of outcomes in planning and governance processes, including lack of reliable data, poor analysis, misinterpretation, and wrong presentation of decision-making outcomes. These barriers may contribute to less transparency and accountability in urban governance [116]. As new data sources are based on complex algorithms, planners and decision-makers do not always have the expertise to use this information by themselves. Local governments cannot afford the costs of collecting, processing, and analyzing big data for immediate decisions. These technical challenges are also imaginable in businesses when it comes to a crisis. While digital technology in businesses is increasingly gaining popularity in everyday situations, managers are not equipped with appropriate skills and resources to mitigate the adverse effects of disturbances such as COVID-19 by employing technology-driven techniques [117]. Therefore, empowering people, business managers, workers, and vulnerable populations for increasing technology literacy is necessary to increase the efficiency and inclusiveness of smart cities.

### 5.5. The Inefficiency of Education and Remote Working

Regarding the deployment of technology to deliver education services, some challenges affect the quality of services. As some institutions have students from different countries and cultures, there might be some language-driven barriers along with other factors of digital inequality [82]. Additionally, some courses require a physical presence in labs and face-to-face contacts that are almost impossible to be approached through e-learning platforms. Additionally, social skills will develop by socializing with other students, which e-learning cannot provide students with. Studies from pre-COVID times have shown that e-learning cannot necessarily consider the differences as students have different learning styles. So, many students with varying styles of learning may fall behind their peers, which will lead to inequality in the education system [118].

Regarding teleworking, it provides the employees with a flexible schedule; however, it may lead to diminished boundaries between private and professional life, causing an imbalance between personal concerns and work duties [119]. However, a survey done by Hu [120] showed that one of the main benefits of remote working is improving work–life balance and this may even lead to improving productivity. Despite this, organizational cultures and morals could be negatively affected by long-term remote working [83]. Moreover, the well-being of workers might be negatively affected due to overwhelming schedules [106]. From a technical lens, remote working has some risks regarding the privacy of information and security of access to data. Hacking websites and platforms is a common challenge of remote working, specifically those jobs which require high security due to their legal and ethical security, confidentiality, and privacy [121].

## 6. Concluding Remarks, Policy Implications, and Future Research Pathways

Technology plays a critical role in today’s disaster management. The COVID-19 experience has shown that the smartness of cities could mitigate urban dysfunctionalities and enhance the well-being of communities. This study has reviewed the literature and focused on empirical cases to show how cities’ smartness and technology deployment have affected the resiliency of the city and the well-being of residents. While technology has improved different stages of crisis management, from an urban planning perspective, its tangible effects have been by: firstly, increasing the participation of residents, enhancing the transparency of governmental processes, and social connectedness; secondly, improving the physical and mental health of residents; and lastly, increasing urban functionality in education and employment systems. The result of our literature review showed that technology deployment in all these three areas has increased the well-being of the community and enhanced the functionality of urban systems. Moreover, our analysis in the COVID-19 era revealed that smart cities are more capable and reliable to improve the well-being of their residents and maintain their functionality during pandemics [55,113,122]. However, there were some challenges and barriers regarding technology deployment in crisis management, including privacy, confidentiality and trust issues, social inclusiveness, political bias and misinformation dissemination, technical issues, and urban functions in the education and employment sectors.

In terms of policy implications, this study revealed that developing smart cities is not achievable by just using technologies to improve urban functions and should also involve considering other aspects and actors such as people and institutions [15,44]. This review also has some policy implications for urban planning practitioners, policymakers, and local government to shed some light on the policy side of digitalization processes. Firstly, policymakers should take technology deployment in cities as a democratic process. People from all social groups have the right to adopt or deny the digitalization of urban functions. Secondly, authorities and policymakers should empower society with non-technical technology deployment issues to increase the applicability and efficiency of these processes, specifically in the education system and regarding organizational implications of remote working. Thirdly, telemedicine could be a sustainable approach for urban planning to facilitate the continuity of everyday situations. Fourth, social media, data gathering, monitoring systems, telemedicine, and tracking applications are pursued by various private, public and sectorial governmental bodies. There is a critical need for integrating these capabilities in the city through promoting integrated digitalization policies.

This study is not an exhaustive literature review of technology deployment in crisis management due to the broadness of the topic and the interdisciplinary nature of the research. So, there are a number of areas in urban planning that could be investigated in future research, including: first, as Papadopoulos, Baltas and Balta [117] discuss, there is a vital need for more research on different aspects of the interaction between technology and socio-cultural background of consumers and users. Second, as Fan, Zhang, Yahja and Mostafavi [122] found in their research, there are algorithms regarding the type of information people trust. Therefore, more research is needed to categorize information sources and clarify how the transparency of governments can affect the credibility of authentic information resources. Third, there is a critical need for more studies regarding the inequality of technology distribution in rural areas to find initiatives and practical solutions. Fourth, the digitalized lifestyle during COVID-19 may become dominant in the post-COVID era and affect the sustainability of urban development by increasing a new wave of suburbanization. So, there is a necessity for urban research to analyze the dynamic of people in metropolitan regions and find policies and strategies to regain the sustainability of urban development under new situations. Finally, it should be acknowledged that only peer-reviewed articles were analyzed for the purpose of this study. It is expected that many other examples of deploying smart solutions for better crises management are reported in grey literature. Therefore, we suggest that future research should also include cases and evidence reported in sources other than peer-reviewed academic literature. Despite these shortcomings, we believe this study is of great interest to reseachers and planners that endeavour to adopt technological solutions for crisis management. It also complements other studies that have examined how smart solutions have contributed to better resilience against the pandemic [123].

## Figures and Tables

**Table 1:** Technology deployment and functions to improve participation, transparency and social connectedness during COVID-19.

Functions	The Type of Technology	Example of Cities Applied These Technologies	Opportunities
Open sharing with citizens about the spread and management of COVID-19	Mobile apps	Seoul	UpCode is making its platform available for others to re-use in other contexts. Retweeting content from other city agencies Predicting outbreaks through interpreting and analyzing social media contents by AI
Sharing Health records and making available for public enforcing social distancing	TraceTogether app The Boston Dynamics Spot robot	Singapore, New York, London, Tel Aviv	
Increase the preparedness and real-time responses	“Crush Aedes Totally” (CAT)	Penang	
Public communication and engagement	Twitter	Atlanta, W.DC	
Using social media to interact with public	WhatsApp	Johannesburg	
For social communication	Facebook	Philippines	
To predict spread of disease	AI	The US cities	

**Table 2:** Technology deployment and functions to improve the physical and mental health of residents during COVID-19.

Functions	The Type of Technology	Example of Cities Applied These Technologies	Opportunities
Mass location tracking of citizens	Digital epidemiological investigation	Tel Aviv	track down potential contacts of infected individuals The improved facial-recognition system allows better tracing and tracking of movement of a COVID-19 person under investigation telemedicine can reduce healthcare inequities for patients in remote areas
Temperature measuring through cameras even with face masks, for potential detection	Next-Generation Artificial Intelligence Development Plan	Wuhan, Shanghai, Beijing, Tokyo	
Self-quarantined Patient monitoring for recording changes in symptoms	“Self-quarantine Safety Protection” smartphone app	Seoul, Singapore	
To promote digital health equity	Telemedicine	New York	
Tracking online shopping products to make assurance	Coronavirus Clearance Certificate (CCC) based on blockchain technology,	Birmingham	
Using dashboard to monitor and predict the spread of the virus, and processing and analysing the data	Smart Control Dashboard	Dubai, New York, London, Berlin, New Castle, Birmingham	
to measure physical distance between people	CCTV and GIS trackers	New Castle	
Using wearable devices to facilitate digital checking and provide information about safe places	COVID-19 contact tracing wearable	Singapore, Tokyo,	
To increase efficiency of restrictions based on big data and simulation	CGA Simulation	Liverpool	
To monitor the mobility of people and track the spread of the virus	HES computer application	Istanbul, San Francisco, Auckland, Milan	
to self-asses their coronavirus risk category	Online COVID-19 Triage Tool	Nigeria, Iran	

**Table 3:** Technology deployment and functions to improve education and employment during COVID-19.

Functions	The Type of Technology	Example of Cities Applied These Technologies	Opportunities
To enable basic needs jobs through delivery systems	food and grocery delivery services, Deliveroo, Peapod, Instacart, or BuyMie	London	if some unwanted emergencies happen in the future, then tourists’ experiences can still be enriched
To enhance tourism industry functions during and post-COVID	robotics, AI and the Internet of Things on service delivery	Chi Minh, Barcelona, Budapest, London	
for an increasingly distributed workforce	provider of network security	Tel Aviv	
enable contract tracing and avoid a full lockdown	Data Hub	Seoul	
To provide smart and creative economic stimulation policies for recovery of jobs	air quality sensor network	London	
Keeping interaction between teachers and students	Facebook	Philippines	
To increase the interconnection between students, parents and teachers	e “Internet + Protocol-guided Learning” teaching model and established public information exchange platforms	Changyuan City, Tehran, Manchester	
